# The Ascidia *Ciona robusta* Provides Novel Insights on the Evolution of the AP-1 Transcriptional Complex

**DOI:** 10.3389/fcell.2021.709696

**Published:** 2021-08-03

**Authors:** Pina Marotta, Federica Salatiello, Luca Ambrosino, Federica Berruto, Maria Luisa Chiusano, Annamaria Locascio

**Affiliations:** ^1^Stazione Zoologica Anton Dohrn, Department of Integrative Marine Ecology, Naples, Italy; ^2^Stazione Zoologica Anton Dohrn, Department of Research Infrastructures for Marine Biological Resources, Naples, Italy; ^3^Stazione Zoologica Anton Dohrn, Department of Biology and Evolution of Marine Organisms, Naples, Italy; ^4^Department of Agriculture, Università degli Studi di Napoli Federico II, Portici, Italy

**Keywords:** Jun, Fos, bZIP protein, mesenchyme, transcription factor, notochord

## Abstract

The Activator Protein-1 transcription factor family (AP-1) transcriptional complex is historically defined as an early response group of transcription factors formed by dimeric complexes of the Jun, Fos, Atf, and Maf bZIP proteins that control cell proliferation and differentiation by regulating gene expression. It has been greatly investigated in many model organisms across metazoan evolution. Nevertheless, its complexity and variability of action made its multiple functions difficult to be defined. Here, we place the foundations for understanding the complexity of AP-1 transcriptional members in tunicates. We investigated the gene members of this family in the ascidian *Ciona robusta* and identified single copies of Jun, Fos, Atf3, Atf2/7, and Maf bZIP-related factors that could have a role in the formation of the AP-1 complex. We highlight that mesenchyme is a common cellular population where all these factors are expressed during embryonic development, and that, moreover, *Fos* shows a wider pattern of expression including also notochord and neural cells. By ectopic expression in transgenic embryos of *Jun* and *Fos* genes alone or in combination, we investigated the phenotypic alterations induced by these factors and highlighted a degree of functional conservation of the AP-1 complex between *Ciona* and vertebrates. The lack of gene redundancy and the first pieces of evidence of conserved functions in the control of cell movements and structural organization exerted by these factors open the way for using *Ciona* as a helpful model system to uncover the multiple potentialities of this highly complex family of bZIP transcription factors.

## Introduction

It is now commonly recognized that the complexity of organisms does not always directly correlate with the number of genes in its genome. Genetic diversity is often ensured by more sophisticated control and interactions among the already available factors (Levine and Tjian, 2003; Van Nimwegen, 2003; Ranea et al., 2005). One of the best ways of creating a wider possibility of regulatory responses is through the formation of transcription factor (TF) dimers, with specific DNA-binding properties, that allows the definition of novel control circuits (Klemm et al., 1998). To understand animal evolution, it is important to delineate how DNA binding and protein interactions of dimerizing TFs evolved. Jun and Fos, belonging to the bZIP proteins, are dimerizing factors found in all eukaryotes and are the most common components of the Activator Protein-1 TF family (AP-1).

The AP-1 complex collectively describes a group of structurally andfunctionally related TFs of the Jun (Jun, JunB, and JunD) and Fosprotein families (Fos, FosB, Fra-1, and Fra-2) and some members of the Atf (Atf, Atf-2, and Atf-3) and Jdp (Jdp-1 and Jdp-2) subfamilies. These factors share structural similarities and form homo- and heterodimeric complexes among them. Each of these proteins is differentially expressed and regulated, determining that every cell type has a complex different mixture of AP-1 dimers with different functions (reviewed by Wagner, 2001). The Jun proteins exist both as homo- and heterodimers, while the Fos proteins form stable heterodimers with Jun and the other AP-1 proteins. All the combinations of AP-1 complexes bind a consensus sequence along the DNA (5′-TGAG/CTCA3′), known as the 12-*O*–tetradecanoylphorbol-13-acetate (TPA)-responsive element (TRE) (Angel and Karin, 1991), and control both basal and inducible transcription of several genes containing the AP-1-binding sites in their regulatory sequences.

During evolution, various layers of AP-1 complexity have appeared such as gene duplications and differences between bZIP members and changes in their co-localization and interaction. This diversification, together with the emergence of different downstream target genes, greatly contributed to extend their mechanisms of action.

About AP-1 functional evolution, in *Drosophila*, Fos is crucial for dorsal closure (Zeitlinger et al., 1997), and in *Caenorhabditis elegans*, it is necessary for cell invasion through the basement membrane during vulvar development (Sherwood et al., 2005). Among vertebrates, specific roles have been assigned to c-Fos and c-Jun in skeletal and bone morphogenesis, where loss of these genes in mice causes osteopenia and defective bone remodeling (Wang et al., 1992; Kenner et al., 2004). In particular, c-Jun is required for axial skeletogenesis by regulating notochord survival and intervertebral disc formation (Behrens et al., 2003), and c-Fos is expressed in both nucleus pulposus cells (Lee et al., 2007) and chordomas (Schwab et al., 2009). Furthermore, several *in vitro* and *in vivo* studies pointed to an important role of the AP-1 complex for hematopoiesis specification in different vertebrate species, such as the mouse (Eferl et al., 1999; Lichtinger et al., 2012; Pereira et al., 2013; Goode et al., 2016; Obier et al., 2016), *Xenopus* (Lee et al., 2012), and zebrafish (Wei et al., 2014; Li et al., 2015). However, none of these studies has completely identified the whole molecular mechanisms responsible for these effects, also because of the number of AP-1 family members and their highly variable dimeric composition, that are also depending on specific cellular contexts.

To date, genetically modified mice harboring genetic disruption and/or transgenic overexpression as well as mutant cells isolated from these animals have represented the major source to understand the regulatory functions of AP-1 subunits. They have highlighted how AP-1 mediates gene regulation in response to a plethora of physiological stimuli, including cytokines, growth factors, and stress signals, in a variety of cellular events involved in normal and neoplastic development, such as proliferation, differentiation, apoptosis, and transformation (Karin et al., 1997). Moreover, as a general rule derived from all studies, the AP-1 family members must be present in a well-defined asset to coordinate and ensure proper development or physiology of the organism.

In light of the great complexity of this family of factors, investigating their function in specific cell types and animal models suitable for transgenesis and reverse genetic approaches could be very useful to broaden the knowledge on their multiple functions and mechanisms of action. *Ciona robusta* is an excellent model for genetic and embryonic studies—thanks to its rapid development, traceable cell lineage, and the availability of an efficient electroporation method for transgenic experiments. Furthermore, its phylogenetic position at the base of vertebrate origin before the 2R duplication and its complete and annotated genomic sequence pose this simple chordate to the forefront of the model organisms available for functional and regulatory studies. Most notably is that *Ciona* possesses the basic developmental features of vertebrates driven by non-duplicated gene families.

The ascidian bZIP factors have been identified and classified by Yamada et al. (2003) in 2003. In this work, all the known human bZIP genes were confirmed to have similarity vs. both the *C. robusta* genome and its cDNA/EST collections. With this analysis, 26 candidate genes were identified representing an almost complete set of bZIP genes in *Ciona*, and among them, one *Jun* gene and one *Fos* gene were identified, respectively (Yamada et al., 2003). Next, Imai et al. (2004) analyzed the expression profile of most of the ascidian TFs during *Ciona* embryonic development. They found that *Jun* and *Fos* are expressed in the B7.7 mesenchyme line at the early gastrula stage and in its descendants (Imai et al., 2004). Afterward, José-Edwards et al. (2011) identified a second Fos member expressed in the mesenchyme from the 110-cell stage and transiently in initial tailbud notochord cells.

Some pieces of information are available in the literature about *Ciona Jun* and *Fos* expression, but no data are reported on the other AP-1 members, and hypotheses on their functions have been formulated only on the basis of phylogenetic similarities with vertebrates (José-Edwards et al., 2011). *Jun* was identified in *Ciona* as a target of the Erk signaling, an evolutionarily conserved key pathway that regulates a wide variety of cellular processes, including proliferation, differentiation, apoptosis, and stress responses under both normal and neoplastic conditions (Kobayashi et al., 2018). All these functionalities are typically controlled by the AP-1 complex in other species, therefore suggesting that *Ciona* is a good model to investigate its functional and regulatory evolution (Amoutzias et al., 2006).

We have here further investigated the AP-1 complex in the ascidian *C. robusta* and analyzed its members’ composition and their expression patterns during embryonic development. We, then, investigated in transgenic embryos the effects of *Jun* and *Fos* notochord-specific overexpression, giving, for the first time, an indication of their functional role in the structural organization of the cells.

## Materials and Methods

### Animals

Adult specimens of *C. robusta* were collected in the Gulf of Taranto, Italy, by handpicking at low depth, and transported in seawater tanks to the facility of Stazione Zoologica Anton Dohrn (SZN). Animals were acclimatized at ∼18°C for 2–3 days in open system tanks and fed every day with a Shellfish Diet 1,800^TM^ Instant Algae^®^. Subsequently, they were exposed to continuous lighting for a few days to accumulate mature gametes and to prevent gamete spawning. Embryos were staged following the developmental timeline established by Hotta et al. (2007).

### Paralog Prediction, Network Construction, and Functional Annotation

*Jun* and *Fos* transcription factor gene IDs (778972 and 778607, respectively) were retrieved from the NCBI Gene partition (Brown et al., 2015). The protein sequences of *C. robusta* assembly GCF_000224145.3 annotation release 104 were downloaded from the RefSeq partition at NCBI (O’Leary et al., 2016). An all-vs.-all similarity search of the entire *C. robusta* protein collection was performed using the BLASTp program of the BLAST package (Camacho et al., 2009), setting an *E*-value cutoff at 10^–3^. Based on the detected similarity relationships, proteins were grouped in networks of paralogs according to the procedure described in Ambrosino et al. (2018) using the Network X package (Hagberg et al., 2008). A filtering step was introduced to select different *E*-value thresholds (e^–10^, e^–15^, and e^–30^) that led to the construction of distinct networks of paralogs at different cutoff settings. A complete updating of the functional annotation of the *C. robusta* protein collection was performed with the software InterProScan (version 5.33) (Jones et al., 2014). UniProt proteins with the InterPro domains related to Jun, Fos, ATF3, and ATF2 were retrieved by keyword search performed on InterPro public database. Sequence similarity searches, using the detected *C. robusta* Jun, Fos, Atf3, and Atf2 protein members as probe queries, were performed by scanning the entire non-redundant protein database (nr) with the BLASTp web resource. Sequences from Tunicata, Cephalochordata, and Hemichordata, together with sequences from the echinoderm *Strongylocentrotus purpuratus* (sea urchin) as out-group, were retrieved. Moreover, sequences from the model species *Homo sapiens*, *Danio rerio*, and *Drosophila melanogaster* as reference species of Vertebrata and Protostomia were retrieved from the NCBI Gene partition. Multiple alignments of the retrieved sequences were performed with MAFFT software v7.397 (default parameters) (Katoh and Standley, 2013). Alignments were cleaned with TrimAl v1.4 (Capella-Gutiérrez et al., 2009) using a 0.25 gap threshold, 0.25 residue overlap threshold, and sequence overlap ranging from 50 to 75%. Maximum likelihood trees were constructed with FastTree v2.1.11 (Price et al., 2010) using default parameters. The visualization of the phylogenetic trees was performed with Interactive Tree of Life (iTOL) v4 (Letunic and Bork, 2019). Pieces of information about the domains contained in the multiple alignments were retrieved through the graphic summary of the NCBI BLAST web portal.

### Isolation of AP-1 Member Transcripts

The full-length coding sequences (CDSs) of *Jun* and *Fos*, corresponding to the *Ciona* transcript models KH.C5.610.v1.A.SL1-1 and KH.C11.314.v2.A.ND1-1, respectively, were amplified by PCR using as template the cDNA derived from mRNA poly(A)^+^ from a mix of *Ciona* embryos at tailbud stages (D’Aniello et al., 2011) and from the *Fos* clone of the Gateway-compatible Unigene collection (gift of Dr. Anna Di Gregorio). Similarly, the *Atf3* full-length cDNA corresponding to the transcript model KH.L5.5.v1.A.ND1-1 and its 3’-untranslated region (UTR) was amplified. Regarding *Maf* transcript (KH.C7.294.v1.A.SL1-1), only the sequence covering the last 1,432 bp of the CDS and the first 446 bp of its 3’-UTR was amplified. All the PCR fragments were cloned in the pCR^®^ II-TOPO vector (Thermo Fisher Scientific) and sequenced. The primers are listed in Supplementary Table 1.

### Construct Preparation

The p*Bra* > *Titf1* construct (Spagnuolo and Di Lauro, 2002) was used to prepare the constructs used for the co-electroporation experiments. It contains 790 bp of the promoter region of *Ciona Brachyury* gene (*Bra*) directing notochord-specific patterns of expression in both primary and secondary lineages (Corbo et al., 1997), the *Ciona* endodermal marker *Titf1* gene, and the SV40 poly-A signal. To generate *Bra* > *Jun* and *Bra* > *Fos*, the *Titf1* gene was removed, by digesting the vector with *Bam*HI/*Mlu*I, and replaced with the *Jun* and *Fos* full-length cDNAs, amplified from their respective pCR^®^ II-TOPO clones (see Supplementary Table 1 for primer sequences).

To generate *Bra* > *RFP* and *Bra* > *LacZ*, the *Bra* promoter was excised from the p*Bra* > *Titf1* construct and inserted into the RFP Zeller vector (Zeller et al., 2006) or in the pBlueScript II KS 1,230 (gift of R. Krumlauf, Stowers Institute, Kansas City, United States), which contains the LacZ reporter gene and SV40 poly-A sequences.

### Transgenesis in *Ciona robusta*

Constructs were electroporated into fertilized eggs as previously described (Locascio et al., 1999). Briefly, *Ciona* eggs were deprived of the chorion and of follicular cells and fertilized. The exceeding sperm was washed out through several passages in filtered seawater (FSW) then immediately transferred in a solution containing 0.77 M mannitol and plasmid DNA. Electroporations were performed in 0.4-cm cuvettes using a Bio-Rad Gene Pulser II at constant 50 V and 500–800 μF.

The transgenic embryos were reared up to early neurula (stage 14), mid tailbud I (stage 21), initial tailbud (stage 17/18), early tailbud I (stage 19), and late tailbud I (stage 23) stages, fixed and treated for subsequent analyses [e.g., whole-mount *in situ* hybridization (WISH), phalloidin staining].

Transgenic experiments for morphological observations of the effects of 10, 20, and 40 μg of *Jun* and *Fos* overexpression in notochord precursors were carried out twice and in biological triplicate. Subsequent transgenic experiments were carried out in biological triplicate and repeated at least seven times. The reported percentages of positive signals correspond to the total of five independent electroporations.

### Embryo Staining and Microscopy

#### LacZ Staining

Bra > LacZ transgene expression was visualized by detection of β-galactosidase activity as previously described (D’Aniello et al., 2011). After staining, embryos were washed in 1 × phosphate-buffered saline (PBS), and imaging capture was made with a Zeiss Axio Imager M1 microscope. A minimum of 100 embryos was analyzed in at least six different electroporations.

#### Phalloidin Staining

Transgenic embryos were fixed for 30 min in MEM-PFA [4% paraformaldehyde (PFA), 0.1 M MOPS pH 7.4, 0.5 M NaCl, 1 mM EGTA pH 8.0, 2 mM MgSO_4_ in H_2_O], then washed four times for 20 min in PBT (1 × PBS-0.01% Triton X-100). They were then incubated at room temperature (RT) in PBT2 + 1:100 Alexa Fluor 635 phalloidin (Thermo Fisher Scientific) for 4 h and rinsed for 5 min in PBT. Afterward, embryos were washed four times for 20 min, alternating PBT and 1 × PBS. Embryos were imaged with a Leica TCS SP8X confocal laser scanning using the HC PL APO CS2 40 × /0.85 dry objective.

### Whole-Mount *in situ* Hybridization

Single WISH and double WISH were carried out as previously described (Christiaen et al., 2009). Briefly, wild-type embryos for *in situ* experiments were obtained by *in vitro* fertilization and fixed at the desired stages in 4% PFA, 0.1 M MOPS pH 7.5, and 0.5 M NaCl at 4°C overnight.

The *Fos* probe was generated from the 63M13 clone found in the *Ciona intestinalis* Gateway-compatible Unigene collection (Beckman Coulter Genomics, Grenoble, France) (gift from Dr. A. Di Gregorio, New York University College of Dentistry, NY, United States). For the synthesis of *Jun*, *Atf3*, and *Maf* probes, their corresponding TOPO-TA clones were used.

The characterization of the mutant embryos by WISH was performed using notochord-, endoderm-, muscle-, and mesenchyme-specific genes chosen for their definite expression pattern on the base of the data reported in ANISEED at the initial tailbud stage (Brozovic et al., 2018). In particular, the following gene models were considered: KH.C3.225 and KH.C13.35 [human Fibrillinn (FBN1) and Chondromodulin (CNMD) orthologs, respectively) for the notochord, KH.L141.45 for the endoderm, KH.C8.859 [human myosin light chain 2 (MYL2) ortholog] for the muscles, and KH.C1.222 and KH.C5.202 [human aldo-keto reductase family 1 member C1 (AKR1C1) and TAL bHLH transcription factor 2 (TAL2) orthologs, respectively] for the mesenchyme. The clones corresponding to all these genes were found in the *Ciona* Gene Collection (Satou et al., 2002) with, respectively, the following IDs: R1CiGC02k18, R1CiGC05o23, R1CiGC02p08, R1CiGC24p06, R1CiGC01b11, and R1CiGC11k01.

A Zeiss Axio Imager M1 was used for embryo image capture. Pictures were edited with Adobe Photoshop CS6, and adjustments, where applied, were only for clarity without affecting any essential part of the image.

## Results

### Searching for AP-1 Gene Members in *Ciona robusta* Genome

The entire protein collection of *C. robusta* has undergone a comprehensive functional annotation to better characterize all the genes classified as bZIP members related to *Jun* and *Fos* (hereafter called AP-1 members). An all-against-all protein sequence similarity search was performed on the entire protein collection of *C. robusta.* This analysis led to the construction of networks of paralogs that were calculated based on three different *e*-value thresholds with the aim to identify sets of paralog proteins based on different similarity cutoffs. The use of more stringent *e*-value cutoffs, indeed, defines a smaller number of paralogy relationships between proteins, obtaining a larger number of networks, when compared to paralog detections that employ less stringent *e*-value thresholds. This permits to define subgroupings of more similar proteins within groups of less similar protein-encoding genes (i.e., in networks of computationally predicted paralogs defined at less stringent *e*-value thresholds).

**FIGURE 1:**
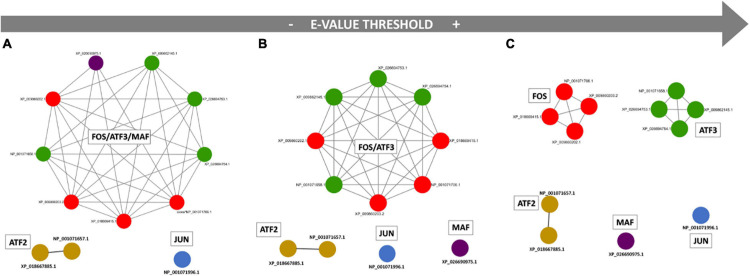
*Ciona robusta* bZIP members related to Jun and Fos when similarity threshold changes. Networks of paralogs were detected at different *e*-value thresholds: **(A)** e^–10^; **(B)** e^–15^; **(C)** e^–30^. Each node in the network represents a protein, and each gray edge represents a paralogy relationship. FOS member isoforms are shown in red, ATF3 in green, ATF2 in light brown, MAF in purple, JUN in blue.

The results of the performed functional annotation analysis were considered in light of the networks of paralog organization in order to identify all those containing the members of the AP-1 family. As represented in Figure 1, members of Fos, Atf3, and Maf (a gene named LOC778711 that in this study we called *Maf* because it encodes a protein with a bZIP Maf motif) are grouped within the same network of paralogs when using the less stringent *e*-value cutoff (e^–10^), while Jun and Atf2 members show a distinct organization. Moving to a more stringent cutoff (*e*-value at e^–15^), the Maf member splits from the Fos/Atf3 group. The use of the more stringent *e*-value threshold (e^–30^), finally, discriminates also Fos from Atf3 members.

A search of all the detected *C. robusta* AP-1 family members in the Gene Partition at the NCBI site revealed that Fos, Atf3, Atf2, Jun, and Maf proteins are isoforms each encoded by a single gene. In particular, we detected four protein isoforms encoded by the *Fos* gene, four isoforms encoded by *Atf3*, two isoforms encoded by *Atf2*, and single proteins encoded by *Jun* and *Maf*, respectively (Supplementary Table 2). A similar search on the considered model species revealed the presence of multiple copies of *Jun* and *Fos* genes in Vertebrata, i.e., three genes related to *Jun* family (*Jun*, *JunB*, and *JunD*) and four genes related to Fos family (*Fos*, *FosB*, *FosL1*, and *FosL2*) in *H. sapiens*; six *Jun* genes (*Jun*, *JunBa*, *JunBb*, *JunD*, *JunDP2a*, and *JunDP2b*) and seven *Fos* genes (*FosAa*, *FosAb*, *FosB*, *FosL1A*, *FosL1B*, *FosL2*, and *FosL2L*) in *D. rerio*. In the considered model from the Protostomia *D. melanogaster*, all the considered AP-1 family members are encoded by a single gene. Details about the number of protein isoforms encoded by these genes are reported in Supplementary Table 2.

To verify the consistency of the results obtained in *C. robusta* and to provide a rough overview of what is available about the AP-1 family members on the reference protein databases, we interrogated InterPro web portal to retrieve all the Uniprot proteins having functional annotation related to Jun, Fos/Atf3/Maf, and Atf2 in all chordate species. By this approach, we obtained 565 different chordate species having 1,993 Jun-related proteins, six of which were protein isoforms from tunicates; 572 different chordate species having 5,505 Fos/Atf3/Maf-related proteins, 10 of which were from tunicates; 511 chordate species having 1,007 Atf2-related protein entries, three of which were from tunicates. All the results are summarized in Supplementary Table 3, and they highlight the widespread presence of AP-1 family proteins among chordates.

The selection of the sequences to be included in the phylogenetic analysis was done by performing sequence similarity searches of the reference NCBI gene sequences of *C. robusta Jun*, *Fos*, *Atf3*, *Atf2*, and *Maf* protein members as probe queries against the entire non-redundant protein database (nr). The probe queries included the reference or the longest isoform for each gene. The best hit for each detected species among the Tunicata, Cephalochordata, and Hemichordata, together with the echinoderm *S. purpuratus* as out-group, was retrieved. The sequences from the model species *H. sapiens*, *D. rerio*, and *D. melanogaster* were selected as reference species of Vertebrata and Protostomia. Most importantly, no significant hit related to the *Maf* probe sequence was detected in the other species, probably revealing a species-specific protein.

The multiple alignments performed with the collected Jun sequences (Supplementary Figure 1) highlighted the presence of the conserved domains “bzip_Jun” (cd14696) and “Jun-like transcription factor” (pfam03957). Accordingly, the retrieved Fos sequences (Supplementary Figure 2) showed the presence of the conserved domain “bZIP_Fos” (cd14721), and the ATF3 and ATF2 sequences (Supplementary Figures 3, 4) highlighted the presence of the conserved domains “bZIP_ATF3” (cd14722) and “bZIP_ATF2” (cd14687), respectively. As expected, all proteins grouped in each alignment showed the presence of the conserved domains “bZIP transcription factor” (pfam00170) and “basic region leucine zipper” (smart00338).

The unrooted phylogenetic tree generated from the collected Jun sequences (Supplementary Figure 5) highlighted the presence of a certain sequence conservation under each taxon. In particular, the following can be easily recognized: a three-leaf branch for Tunicata followed by a cluster of seven leaves for Vertebrata; a three-leaf branch including Cephalochordata and single leaves of Echinodermata, Hemichordata, and Protostomia sequences. The only exception to the clusterization of sequences from the same taxon is represented by sequences from the tunicate *Oikopleura dioca* and zebrafish (*D. rerio*), i.e., the two Jun dimerization proteins 2 (JUNdp2a and JUNdp2b). The unrooted phylogenetic tree generated from the collected Fos sequences (Supplementary Figure 6) showed a similar behavior. Again, we observed a Tunicata branch (with two leaves) related in the phylogenetic tree to a cluster of Vertebrata sequences including multiple copies from human and zebrafish and to a three-leaf branch of Cephalochordata. Also in this case, we can observe for *Oikopleura* and for the FOSL2L gene from zebrafish a position in the tree that is distant from other tunicate or vertebrate sequences and more closely related to leaves of Hemichordata, Echinodermata, and Protostomia (Supplementary Figure 6).

Since we were not able to detect in other species robust homologs of the *C. robusta Maf* gene, we decided to include the sequence of its encoded protein in the phylogenetic analysis related to another member of the AP-1 family. As inferred by our paralog analysis performed on the entire *Ciona* protein collection, *Maf* resulted in the same network of paralogs of *Fos*- and *Atf3*-related protein isoforms when using the less stringent *e*-value cutoff (Figure 1). We tried to perform a new phylogenetic tree including *Maf* protein sequence among *Atf3* and *Fos* sequences. Only the set of *Atf3* sequences enabled the conservation of *Maf* sequence in the multiple alignment after the trimming step. This was probably due to the marked difference between the lengths of *Maf* and *Fos* sequences. The tree generated from the retrieved *Atf3* sequences, including the *Maf* sequence from *C. robusta* (Supplementary Figure 7), showed the presence of three main ATF3 branches with Tunicata ATF3 sequences rather distant from the rest of the considered sequences that are, respectively a two-leaf branch of Vertebrata close to a two-leaf branch of Cephalochordata and a second branch with leaves of sequences from Protostomia, Echinodermata, and Hemichordata. The position of *C. robusta Maf* sequence between Cephalochordata and *D. melanogaster* in the phylogenetic tree probably indicates an ancestral separation of this sequence that soon diverged from the other related sequences of the *ATF3* family. The tree generated from the collected *Atf2* sequences (Supplementary Figure 8) showed a three-leaf branch of tunicates close to a two-leaf branch including *Oikopleura dioica* and *D. melanogaster*. More distant in the tree, we can observe the presence of *Atf2* sequences from Hemichordata and Echinodermata, followed by the considered Cephalochordata and Vertebrata taxa.

The phylogenetic analysis revealed in all the four considered classes an ancestor behavior of AP-1 members of Tunicata subphylum with respect to the other considered sequences. An exception to this behavior is represented by the sequences from *Oikopleura*, a tunicate with peculiar genomic features that make it closer to Protostomia in the phylogenetic trees rather than other tunicates (Supplementary Figures 5, 6, 8). The performed analyses highlighted, moreover, the presence of single gene copies in tunicates opposed to gene amplification of *Jun* and *Fos* members in Vertebrata subphylum (Supplementary Figures 5, 6). Zebrafish in particular has undergone extra-duplication events of its genome, as resulted from the number of copies of Jun and Fos genes (Supplementary Table 2).

### Expression of AP-1 Members During Embryonic Development

**FIGURE 2:**
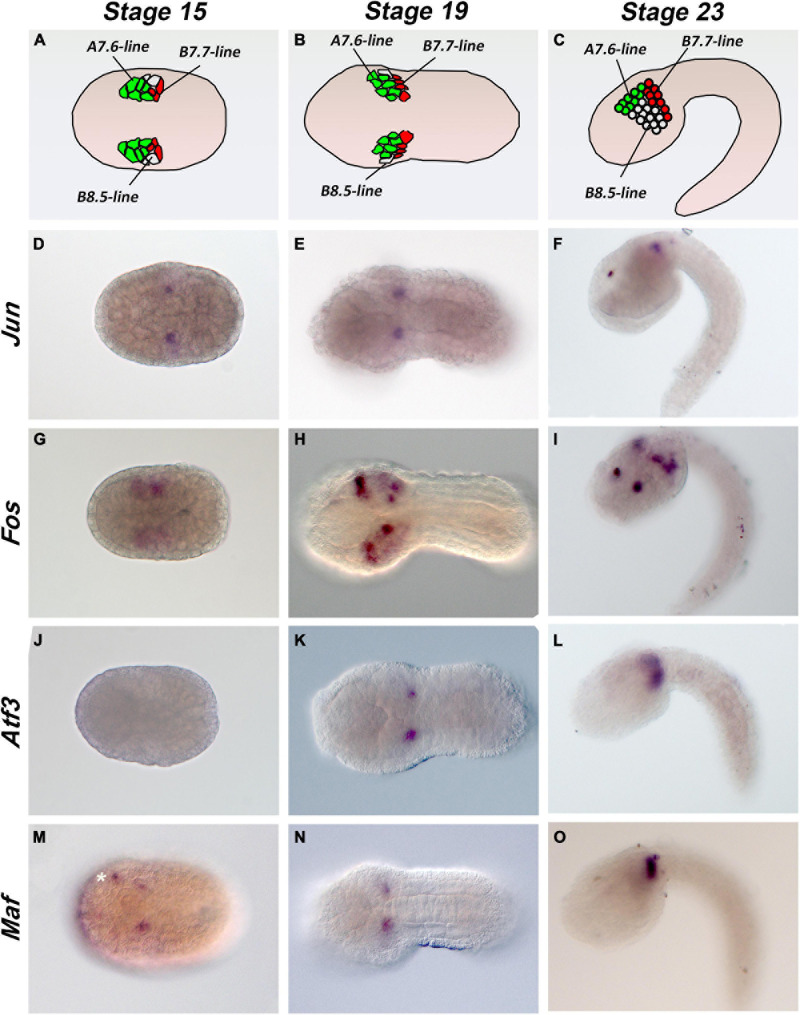
Expression patterns of *Ciona* AP-1 transcription factors during embryonic development. **(A–C)** Schematic representation of mesenchymal cells in stage 15, 19, and 23 embryos. **(D–O)** Whole-mount *Ciona* embryos hybridized *in situ* with antisense RNA probes against the *Jun* **(D–F)**, *Fos* **(G–I)**, *Atf3* **(J–L)**, and *Maf* **(M–O)** genes, as indicated on the left side of each row. Developmental stages are indicated at the top of each column: stage 15, early neurula; stage 19, early tailbud I; stage 23, late tailbud I. White asterisk indicates an unspecific signal. Stage 15 and 19 embryos are in a dorsal view, and stage 23 embryos are in a lateral view; anterior is on the left.

Few data on *Ciona* Fos and Jun factors are currently available in the literature. We hence proceeded with a comparative analysis of the expression profiles of the AP-1 gene members during the embryonic development at neurula, early tailbud, and late tailbud stages (stages 15, 19, and 23, respectively). We assayed by WISH *Jun*, *Fos*, *Atf3*, and *Maf* expression, except for *Atf2/7* of which we were unable to amplify a valid riboprobe. As shown in Figure 2, we obtained very similar expression profiles in specific mesenchymal populations. As already reported in ANISEED database^1^ and by Imai et al. (2004), *Jun* is expressed in a pair of B-cell mesenchymal cells from neurula to tailbud stages (Figures 2D–F); the same narrow signal is detectable for *Atf3* and *Maf* (Figures 2J–O), although *Atf3* appears later at tailbud stage 19. According to single-cell transcriptomic analyses (Cao et al., 2019), *Fos* staining shows a relatively stronger and broader signal in the mesenchymal cells at neurula and tailbud stages (Figures 2G,H) with a very transient signal in notochord precursors at the neurula stage (José-Edwards et al., 2011; data not shown). A novel, not previously reported, and symmetric *Fos* signal is clearly visible in the anterior part of the nervous system at late tailbud I stage (Figure 2I).

### Functional Analysis of *Jun* and *Fos* Transcription Factors

Since our WISH experiments show an overlapping profile of the ascidian AP-1 gene expression, their heterodimerization faculty might be postulated. To corroborate this hypothesis, we focused our functional analysis on Jun and Fos members. The Jun proteins exist as homodimers and heterodimers, while the Fos proteins are historically considered able to form stable heterodimers with Jun proteins, although it has been demonstrated that, in peculiar conditions, they can also homodimerize. We, hence, assayed their activity by overexpression of their CDSs during notochord development under the control of the *Brachyury* promoter (Bra > Jun, Bra > Fos constructs). This tissue-specific expression would permit to avoid possible interference by other members of this family, and it is already reported in other species as a territory with functional significance for Jun and Fos activity.

**FIGURE 3:**
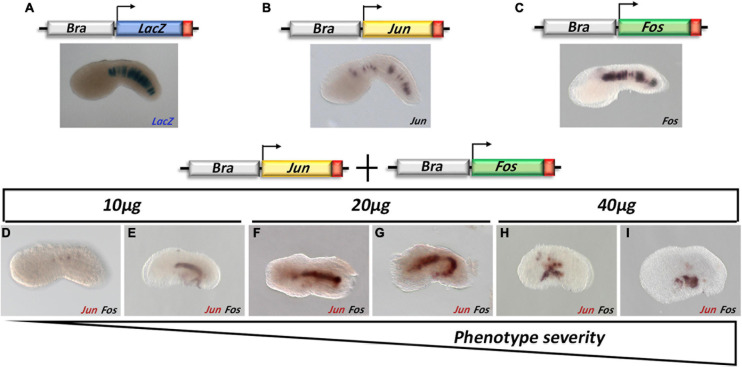
Functional analysis of *Jun* and *Fos* during *Ciona robusta* development. **(A)** Brachyury promoter (Bra) specifically drives the expression of a transgene in the notochord cells; fertilized eggs electroporated with Bra > *LacZ* **(A)**, Bra > *Jun* **(B)**, and Bra > *Fos* **(C)** and reared up to the mid tailbud I stage (S21) were checked for the transgene expression through β-galactosidase assay staining (Bra > *LacZ*) or whole-mount *in situ* hybridization (WISH) using *Jun* (brown staining)- and *Fos* (black staining)-specific probes. **(D–I)** *Jun* and *Fos* double WISH on tailbud embryos co-electroporated with Bra > *Jun* and Bra > *Fos*. Increasing amounts of Bra > *Jun* and Bra > *Fos* go at the same pace with the growing severity of the phenotype; double WISH experiments show that the transgenes are less expressed in the most wt-like embryos **(D)**. For all embryos, the anterior is on the left and the dorsal on the top.

Different concentrations of Bra > Jun and Bra > Fos constructs (10, 20, and 40 μg) alone or in combination were assayed by electroporation. The transgenic embryos were reared up to the mid tailbud I (stage 21), following the notochord cell intercalation, and Jun and Fos overexpression was evaluated by WISH. Compared to the control (*Bra* > *LacZ*), the overexpression in the notochord cells of *Jun* and *Fos* alone apparently did not influence the proper development of the embryos (Figures 3A–C).

Conversely, *Jun* and *Fos* co-electroporation resulted in a scale of abnormal phenotypes proportional to the amount of DNA used (Figures 3D–I) and ranging from “wt-like” in which all the structures of the embryo were perfectly recognizable (Figure 3D) to a completely aberrant one (Figures 3H,I).

Intra-electroporation variability was probably also linked to the stochastic nature of the method, where the variable amount of DNA that enters the fertilized eggs during electroporation is random and unpredictable. Within this scenario, as the quantity of electroporated DNA increases, the percentage of severely defective embryos increases too (Table 1). Indeed, WISH analyses on these embryos revealed that *Jun* and *Fos* expression in notochord cells was barely detectable in the wt-like tailbuds. Hence, depending on the level of overexpression induced in the notochord cells, three groups of embryonic phenotypes have been distinguished (Table 1): a first, almost normal, group of embryos with a nearly perfect development (Figure 3D); a second, moderately defective, group specifically displaying various tail alterations (Figures 3E,F); and finally, a third, severely defective, group exhibiting a deep displacing of the notochord and severe alterations throughout the whole embryo (Figures 3G–I and Table 1). It is worth noting that the development of Bra > *Jun* + Bra > *Fos* embryos proceeds as in control embryos up to the neurula stage, although *Brachyury* promoter starts to be active at the 64-cell stage (Corbo et al., 1997). Therefore, only the formation of tailbud embryos is affected, that is, in correspondence to notochord elongation and cell intercalation stage.

**TABLE 1:** Percentages of phenotypic alterations as a function of the amount of Bra > Jun and Bra > Fos electroporated DNA.

***Bra* > *Jun* + *Bra* > *Fos***	**Normal**	**Moderately defective**	**Severely defective**
10 μg	30%	60%	10%
20 μg	20%	35%	45%
40 μg	2%	38%	60%

Together, these results confirm the ability of the Jun/Fos heterodimeric complex to influence notochord development and seem to indicate that, in *Ciona*, this complex can influence the transcriptional regulation of developmental genes.

### Characterization of Bra > *Jun* and Bra > *Fos* Transgenic Tailbud Embryos

Bra > *Jun* and Bra > *Fos* overexpression in the notochord precursor cells induces progressively more severe alterations with increasing Jun and Fos concentrations. We, then, evaluated the differentiation levels of the various embryonic tissues on transgenic tailbud embryos (stage 17/18) electroporated with 10 μg of Bra > Jun and Bra > Fos DNA and split out in three separate groups according to their apparent phenotype severity. We assayed by WISH experiments the expression of muscle-, mesenchyme-, endoderm-, and notochord-specific markers selected from the ANISEED database for their definite and specific expression pattern (Brozovic et al., 2018).

#### Muscles and Mesenchyme

**FIGURE 4:**
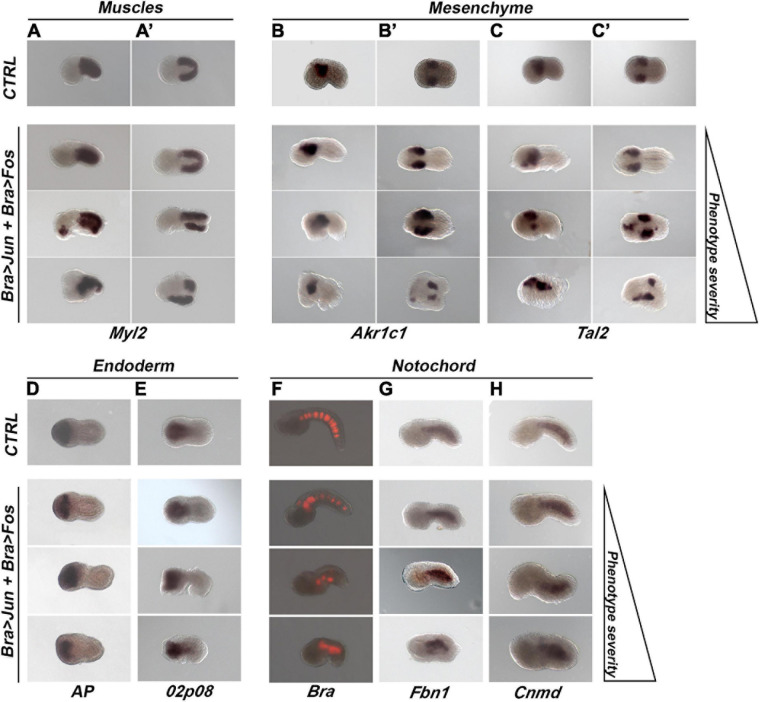
Tissue differentiation in transgenic embryos (stage 17/18) co-electroporated with 10 μg of Bra > *Jun* and Bra > *Fos* constructs. **(A,A’)** Muscle differentiation; whole-mount *in situ* hybridization (WISH) experiments with the muscle marker gene *Myosin Light Chain 2* (*Myl2*). **(B–C’)** Mesenchyme differentiation; WISH experiments with mesenchyme markers **(B,B’)** *Aldo-Keto Reductase Family 1 Member C1* (*Akr1c1*); **(C,C’)** *TAL BHLH Transcription Factor 2* (*Tal2*). **(D,E)** Endoderm differentiation. **(D)** Alkaline phosphatase (AP) assay to detect the endogenous AP enzymatic activity. **(E)** WISH experiment with the endoderm-specific gene KH2012:KH.L141.45. **(F–H)** Notochord differentiation. **(F)** Fluorescent images showing reporter gene [red fluorescent protein (RFP)] expression under the control of the *Brachyury* (*Bra*) promoter in embryos tri-electroporated with 10 μg of Bra > *Jun*, Bra > *Fos*, and Bra > RFP. **(G,H)** WISH experiments with the notochord markers **(G)** *Fibrillin 1* (*Fbn1*) and **(H)** *Chondromodulin* (*Cnmd*). CTRL, Bra > RFP control embryos; Bra > Jun + Bra > Fos, co-electroporated embryos. In each column, embryos have been placed according to the progressive severity of the phenotype: normal (top), mild (middle), and severe (bottom) alterations. For all embryos, the anterior is on the left. **(A–C,F–H)** Lateral view; **(A’,B’C’,D,E)**, dorsal view.

The overexpression of *Jun* and *Fos* in notochord cells appeared not to affect the differentiation of muscle and mesenchymal cells, as far as could be seen by examination, in the manipulated tailbud embryos of the global expression of the muscle-specific gene, *Myosin Light Chain 2* (*Myl2*) (Macera et al., 1992; Figures 4A,A’), and of two mesenchyme-specific genes, *Aldo-Keto Reductase Family 1 Member C1* (*Akr1c1*) (Ciaccio and Tew, 1994; Figures 4B,B’) and *TAL BHLH Transcription Factor 2* (*Tal2*) (Xia et al., 1991; Figures 4C,C’).

#### Endoderm

To assess the correct endoderm differentiation, control and co-electroporated tailbuds were tested for the expression of two endodermal markers, the alkaline phosphatase (*AP*) and a gene, not yet annotated, identified with the ID KH2012:KH.L141.45, that here we refer to as *02p08*. The first was evaluated through an AP activity assay (Figure 4D), the other one by WISH (Figure 4E). These tests showed that the Bra-directed overexpression of *Jun* and *Fos* did not affect the differentiation and localization of the endodermal cells.

### Bra > *Jun* and Bra > *Fos* in Notochord Organization

The effects of the overexpression of *Jun* and *Fos* on the differentiation of notochord cells were first explored by WISH of the expression of two different markers exclusively expressed in this tissue: *Fibrillin 1* (*Fbn1*) (Sakai et al., 1986) and *Chondromodulin* (*Cnmd*) (Shukunami and Hiraki, 2001; Figures 4G,H). Furthermore, the activity of the *Bra* promoter was also assayed by tri-electroporation with Bra > *Jun* and Bra > *Fos* of the Bra > *RFP* construct containing the red fluorescent protein (RFP) downstream of the *Bra* promoter (Corbo et al., 1997; Figure 4F). From these analyses emerged that the expression of the examined notochord markers shows the same pattern as in the controls independently from their phenotype severity and that notochord cell differentiation is not affected. Regardless of the grade of progressive disorganization in the different phenotypes, by terminal deoxynucleotidyl transferase dUTP nick end labeling (TUNEL) assay, we also observed that notochord alterations were not caused by the induction of apoptosis (data not shown) and that the number of notochord cells was comparable to that of the controls. These results suggest that the Jun and Fos could be involved in the structural organization of the notochord but not in its specification, proliferation, and differentiation.

**FIGURE 5:**
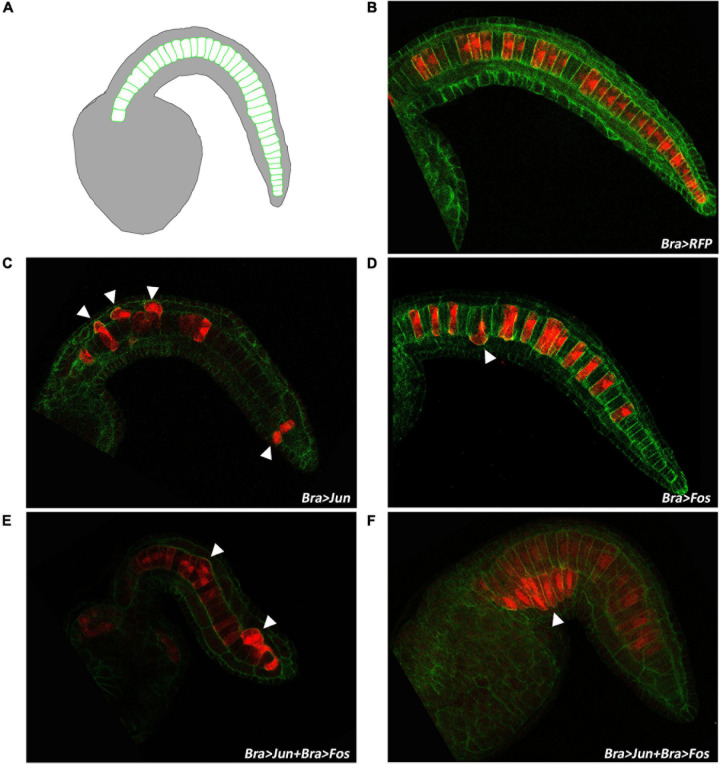
Intercalation defects in tailbud embryos overexpressing *Jun* and *Fos*. **(A)** Schematic representation of notochord cell organization in the late tailbud embryo (stage 23). **(B)** Confocal microscopy of a stage 23 control embryo electroporated with Bra > RFP. **(C,D)** Stage 23 embryos co-electroporated with Bra > RFP and either Bra > Jun or Bra > Fos, respectively. **(E,F)** Embryos at the same stage, co-electroporated with Bra > RFP and Bra > Jun plus Bra > Fos showing altered organization of notochord cells along the tail. White arrowheads evidence misaligned cells. Red, red fluorescent protein (RFP) staining; green, phalloidin staining.

To deeply explore the effects of Bra > *Jun* and Bra > *Fos* expression on the notochord morphology, transgenic embryos were left to develop until the late tailbud I stage (stage 23), when the notochord cell intercalation is completed, but before lumen formation (Figure 5A), and then, the transgenes were observed under a fluorescence microscope (Figures 5B–F). For this analysis, we again used 10 μg of Bra > *Jun* and Bra > *Fos* alone or in combination and added in all assays the Bra > RFP construct as an internal control to selectively evaluate the correct formation of electroporated notochord cells. Furthermore, phalloidin staining of the embryos was used to define cell membrane profiles of the notochord. Number counting of RFP-positive embryos evidenced a notable decrease, almost by half, of developed embryos among those electroporated with Jun and Fos together (Supplementary Table 4). As annotated in Table 2, the electroporation efficiencies, evaluated based on Jun/Fos embryo RFP expression, were comparable in all samples, ranging from 83.15% of the control Bra > RFP embryos to 92.31% of the tri-electroporated. Among the fully developed embryos, we then evaluated notochord impairment and considered normal only the notochords with a perfect alignment of all cells. Following electroporation of the Bra > RFP construct, control larvae displayed the wild-type notochord phenotype (Figures 5A,B). In *Jun* and *Fos* transgenic embryos, we observed two types of statistically significant alterations in notochord cells. In particular, about 11% of Bra > Fos and 23% of Bra > Jun embryos displayed single notochord cell misalignments (Figures 5C,D). This incorrect positioning of single notochord cells increases to 30% in Bra > Jun plus Bra > Fos co-electroporated embryos (Table 2). Notably, a second more severe misalignment of notochord cells was mostly specific for *Jun* and *Fos* co-electroporated samples. In 29% of *Jun/Fos* embryos, we observed a displacement of groups of notochord cells (Figures 5E,F). This wider notochord disorganization has always been identified when no less than 4–5 consecutive cells were electroporated as demonstrated by their RFP-positive signal (Figures 5E,F). Conversely, we never observed single or in blocks misalignments in the notochord when there were no consecutive cells electroporated. Cell misalignment equally involved anterior and posterior notochord cells (Figures 5E,F), thus revealing an equal impact of Jun and Fos on notochord cells deriving from primary and secondary lineages, respectively.

**TABLE 2:** Percentages of notochord alterations observed in Bra > Jun and Bra > Fos transgenic embryos.

**Constructs**	**Electroporation efficiency %**	**Fully developed embryos N.**	**Notochord phenotypes**		
			**Normal (%)**	**Single cell alteration (%)**	**Blocks of cells alteration (%)**
BRA > *RFP*	83.15%	178	67.98%	2.70%	0.68%
BRA > *Fos*	91.35%	104	72.11%	11.58%	1.05%
BRA > *Jun*	88.55%	131	62.93%	23.27%	6.03%
BRA > *Jun* + BRA > *Fos*	92.31%	104	32.29%	30.21%	29.17%

The 23% of single-cell alterations in embryos electroporated with *Jun* might be easily explained if we consider the homodimerization ability of this TF. Conversely, 11% of misalignments in embryos overexpressing *Fos* could be presumably the consequence of the formation of Fos homodimers or heterodimers with unknown factor/s. Whichever is the most correct hypothesis, these complexes seem in any case less effective than Jun/Fos combined activity, and further studies will be necessary to verify if the phenotype derives from the formation of Jun–Fos heterodimers.

In particular, the observed partial convergence of notochord cells to form two adjacent and opposing rows (Figures 4, 5E,F) is very similar to the knockdown phenotype of *Fibronectin* (*Fn*) gene (Segade et al., 2016). *Fn* gene is required for proper notochord intercalation in *Ciona* and, as well as in *Jun/Fos* transgenic embryos, its functional perturbation does not disrupt sheath integrity and defective cells do not escape into adjoining tissues (Figures 5E,F). Given this similarity in phenotypes, to assess if *Jun* and *Fos* co-expression can affect notochord intercalation through the negative regulation of *Fn*, we analyzed the expression of this gene by WISH on transgenic embryos (Supplementary Figure 9). The result was that *Fn* expression does not change in any of the embryos with *Jun* and *Fos* ectopic expression alone or in combination (Supplementary Figures 9B–E). *Fn* expression remains unaltered even in severely defective phenotypes in which the notochord cells did not intercalate and are still placed in two separate rows (Supplementary Figure 9E).

Taken together, these data suggest that the overexpression of *Jun* and *Fos* affects notochord cell intercalation but not through *Fn* regulation.

## Discussion

Despite all the existing data, it is difficult to draw a clear picture about the general physiological role for the AP-1 transcriptional complex in cellular processes and in the embryonic development. Based on the literature, it is quite obvious that the abundance of different AP-1 members within given cell types, as well as cell lineages, differentiation stage, microenvironment, and type of stimulus, has a large impact on how AP-1 modulates the determination of cells to proliferate, differentiate, or die by apoptosis.

To better characterize in *C. robusta* the predicted paralogs of the AP-1 family members and their evolutionary relationships, we searched for sequence similarities in the NCBI and Uniprot databases and performed phylogenetic analyses of the retrieved sequences.

The genomic locus assignment of each protein sequence revealed the presence of single gene copies for each *Ciona* AP-1 factor. In particular, single Jun, Fos, Atf2, and Atf3 members and an additional *Ciona*-specific Maf member evidenced less redundancy for this bZIP family in ascidians and the presence of a highly conserved core of AP-1 members that was formed in the last common ancestor of chordates.

Individual members of each family may have different biological functions during the formation of the embryonic tissues. By comparative analysis of these gene expression profiles during *Ciona* development, it was evident that they all show a common and restricted pattern in the B7.7 mesenchyme line (Figure 2). In *C. robusta* and *savigny*, it was demonstrated that this line gives rise to tunic and blood cells (Tokuoka et al., 2005). In this regard, different *in vitro* and *in vivo* molecular genetic approaches have demonstrated the many roles of AP-1 (Fos/Jun) TF in the development and differentiation of hematopoietic precursor cells (reviewed in Liebermann et al., 1998). However, none of these studies has completely identified the whole molecular mechanisms responsible for these effects also because of the number of AP-1 family members and their highly variable dimeric composition. The presence of single gene copies for *Ciona Fos*, *Jun*, *AT3*, and *Maf* factors and their common expression in embryonic mesenchyme cells that will give rise to blood cells reveal interesting potentialities about the different combinations of dimeric factors they can form and their possible conserved function in hematopoiesis. In this regard, ascidians with their not duplicated number of AP-1 members may represent an excellent experimental system to explore the evolution of AP-1 mechanisms of action in hematopoiesis.

A wider *Fos* expression profile, not only in mesenchymal cells but also in notochord and neural precursors of the sensory vesicle, indicates that Fos may have functions that are Jun/Atf/Maf-independent and suggests that it may form homodimers or heterodimers with other than AP-1 factors (Szalóki et al., 2015).

Although we still do not know which are the partners of Fos in the notochord and its mechanism of action, our results after *Jun* and *Fos* ectopic expression in notochord cells evidenced that Fos and Jun alone can induce narrowed alterations limited to single cells, while only the combined presence of both factors induces structural disorganization and severe impairment of cellular intercalation. This result is perfectly in agreement with the expression of *Fos* only in the initial differentiation of the notochord cells and evidenced how important is its downregulation so that correct intercalation can occur. Further indirect evidence about the presence of AP-1 consensus motif in notochord *cis*-regulatory modules that are able to cooperate with Foxa2 in activating the expression of notochord-specific genes (José-Edwards et al., 2015) contributed to strengthening our conclusions. Our findings are in support of a conserved and yet unexplored genetic program regulated by Fos in notochord formation that additional information on *Ciona* AP-1 mechanisms of action could help clarify. Similarly, a fundamental role played by AP-1 complex was evidenced in vertebrates for axial skeletogenesis, for proper histo-architecture of the epidermis, and for mesenchymal–epithelial cross-talk in the skin (Angel and Szabowski, 2002; Behrens et al., 2003). Interestingly, Jun/Fos-induced alterations in notochord cells clearly resemble the phenotypes observed when the planar cell polarity (PCP) process is impaired. In vertebrates, the PCP pathway is important for developmental processes in a number of organs and tissues as in the control of convergence and extension during gastrulation and in the control of the cell behaviors that drive notochord intercalation (Wallingford et al., 2000; Topczewski et al., 2001). Interfacing the results obtained in ascidians and vertebrates on the PCP pathway and its connection with the AP-1 complex, interesting hypotheses of functional conservation can be applied. Key components mediating cell–cell communication in the PCP vertebrate signaling pathway include the Wnt non-canonical pathway, the transmembrane protein Frizzled (Fz), and the cytoplasmic proteins Prickle (Pk) and Disheveled (Dsh), which culminate in the activation of c-Jun N-terminal kinase (JNK) and of the AP-1 complex to promote actin cytoskeleton reorganization and cellular movements. Comparatively, the ascidian Wnt5, Prickle, and Dsh mutants result in loss of PCP signaling and of notochord cell intercalation in the mediolateral axis (Keys et al., 2002; Jiang et al., 2005; Niwano et al., 2009). In light of this conserved PCP pathway, it will be highly interesting to investigate the involvement of the AP-1 transcriptional complex in the ascidian PCP pathway and its role in axis elongation and cellular intercalation. To this aim, it will be fundamental to identify the other putative AP-1 members responsible together with Fos for notochord correct development.

## Conclusion

The number of proteins forming the AP-1 complex and their variable combination in specific tissues always strongly impaired the ability to clarify their mechanism of action during normal and neoplastic development. In this study, we evidenced the presence of non-duplicated AP-1 gene members and placed the foundations for understanding the complexity of the AP-1 transcriptional family during embryonic development in ascidians.

We contributed to understanding their potential function in controlling the correct structural organization of cells. Full comprehension of *Ciona* AP-1 transcriptional complex and of its role in mesenchyme differentiation and notochord intercalation could represent an important turning point to understand their direct function in vertebrate erythropoiesis and notochord formation.

Further comparative studies among ascidians and other chordates about AP-1 functional role in PCP movements could reveal interesting similarities as well as novel and significant differences in the mechanisms underlying chordate axis elongation.

## Data Availability Statement

Publicly available datasets were analyzed in this study. This data can be found here: http://www.aniseed.cnrs.fr/.

## Author Contributions

PM, FS, and FB did the molecular experiments. LA did the bioinformatics analyses. PM, FS, LA, and AL drafted the manuscript and prepared the figures. AL supervised the experiments and edited and revised the manuscript. MC supervised the bioinformatics analyses and revised the manuscript. All authors approved the manuscript for publication.

## Conflict of Interest

The authors declare that the research was conducted in the absence of any commercial or financial relationships that could be construed as a potential conflict of interest.

## Publisher’s Note

All claims expressed in this article are solely those of the authors and do not necessarily represent those of their affiliated organizations, or those of the publisher, the editors and the reviewers. Any product that may be evaluated in this article, or claim that may be made by its manufacturer, is not guaranteed or endorsed by the publisher.

## Footnotes

1
http://www.aniseed.cnrs.fr/
