# Supplementary figures and images for: The Ascidia Ciona robusta Provides Novel Insights on the Evolution of the AP-1 Transcriptional Complex
Source: Front Cell Dev Biol. 2021 Aug 3;9:709696. doi: 10.3389/fcell.2021.709696 (PMC8369891; doi:10.3389/fcell.2021.709696)

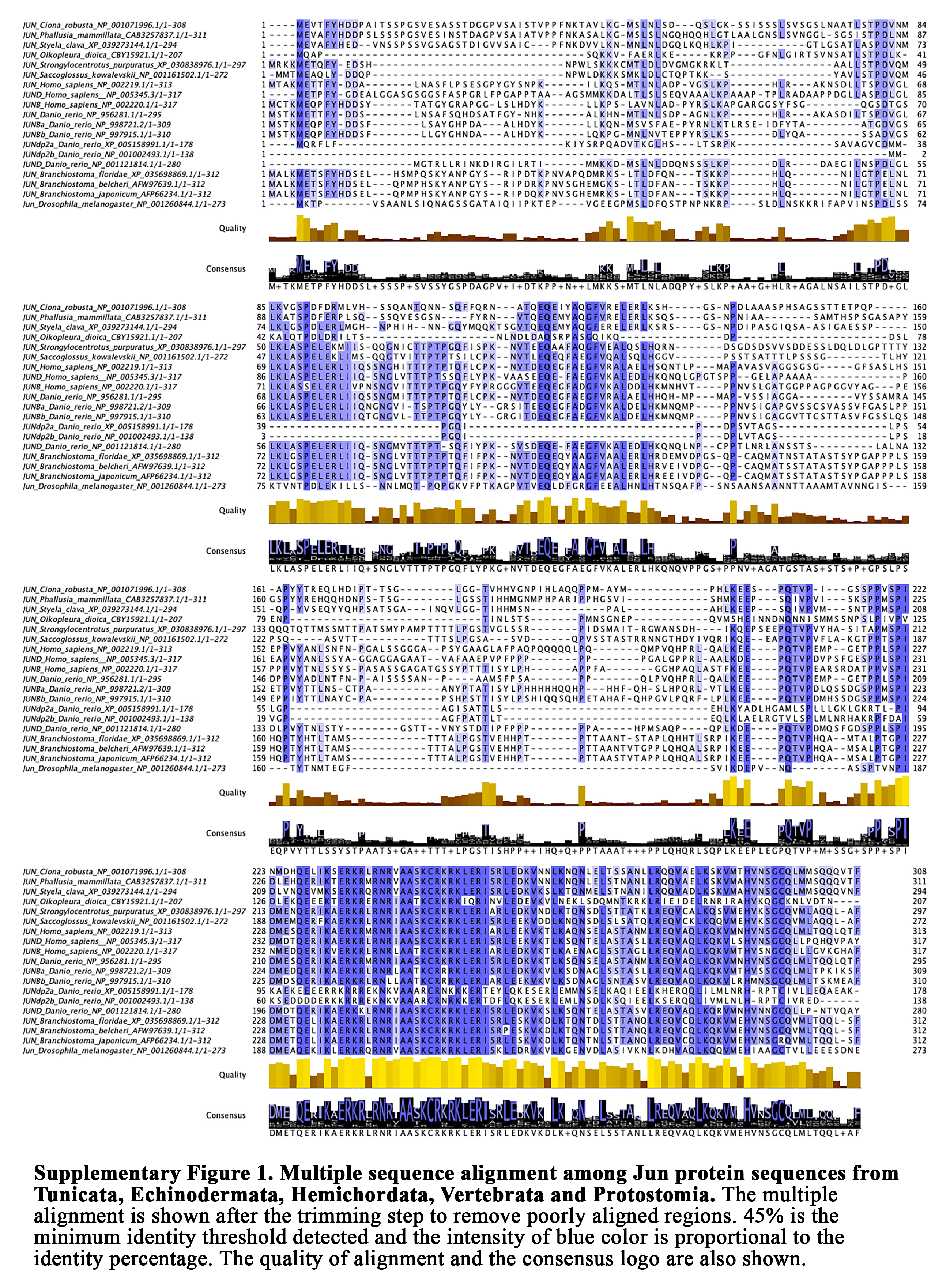

Supplement: Supplementary file 5 [file Image_1.TIF]

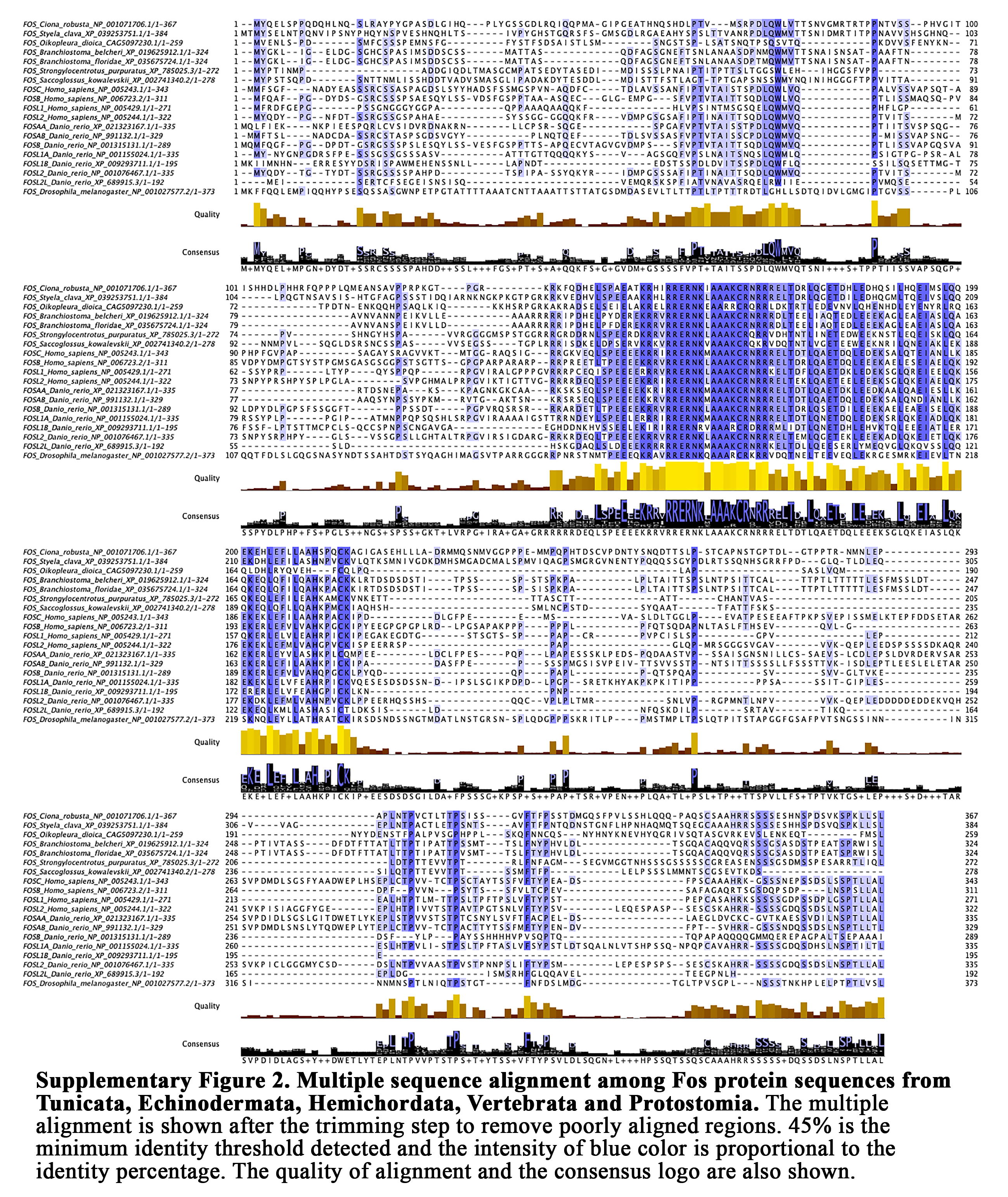

Supplement: Supplementary file 6 [file Image_2.TIF]

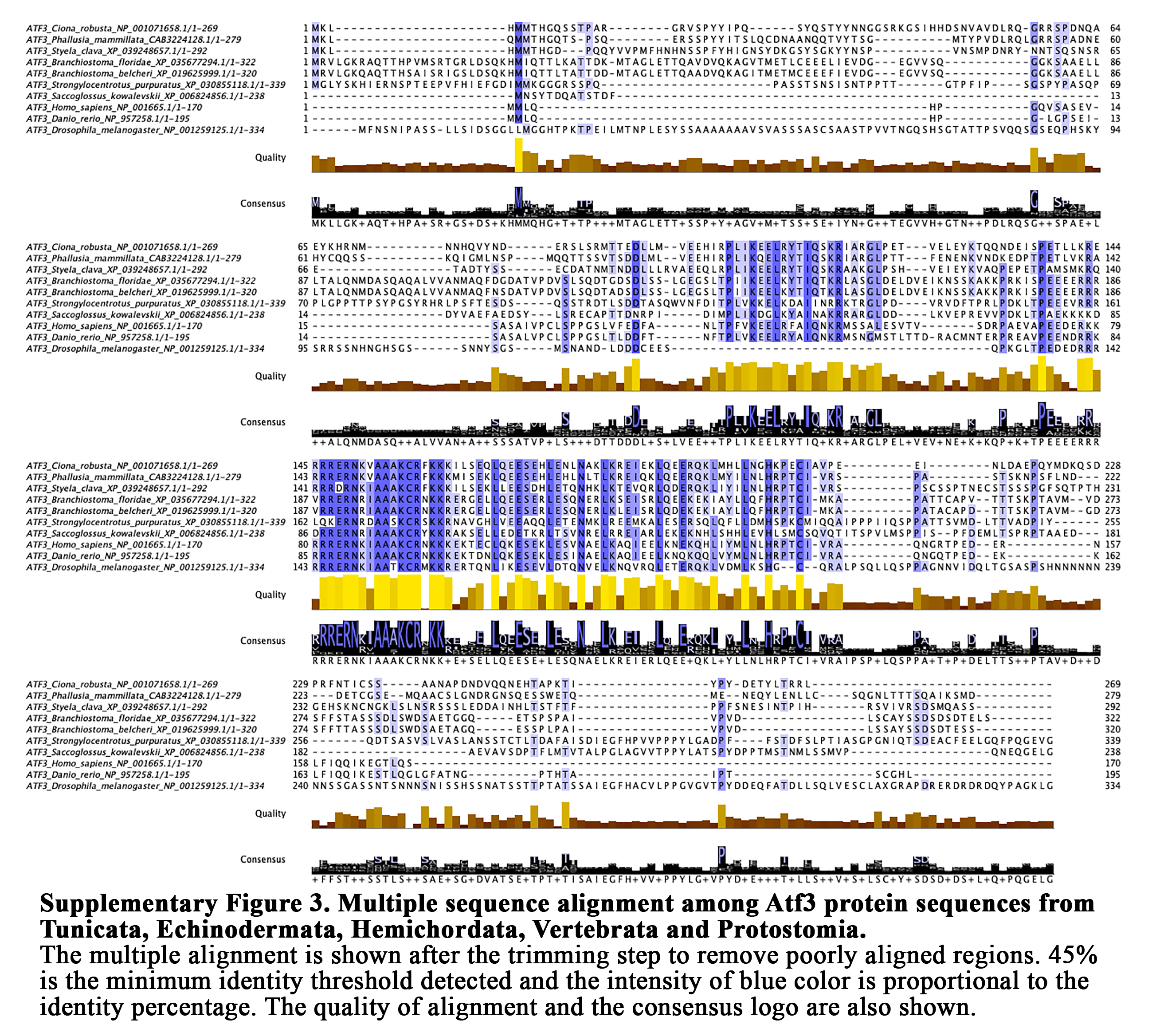

Supplement: Supplementary file 7 [file Image_3.TIF]

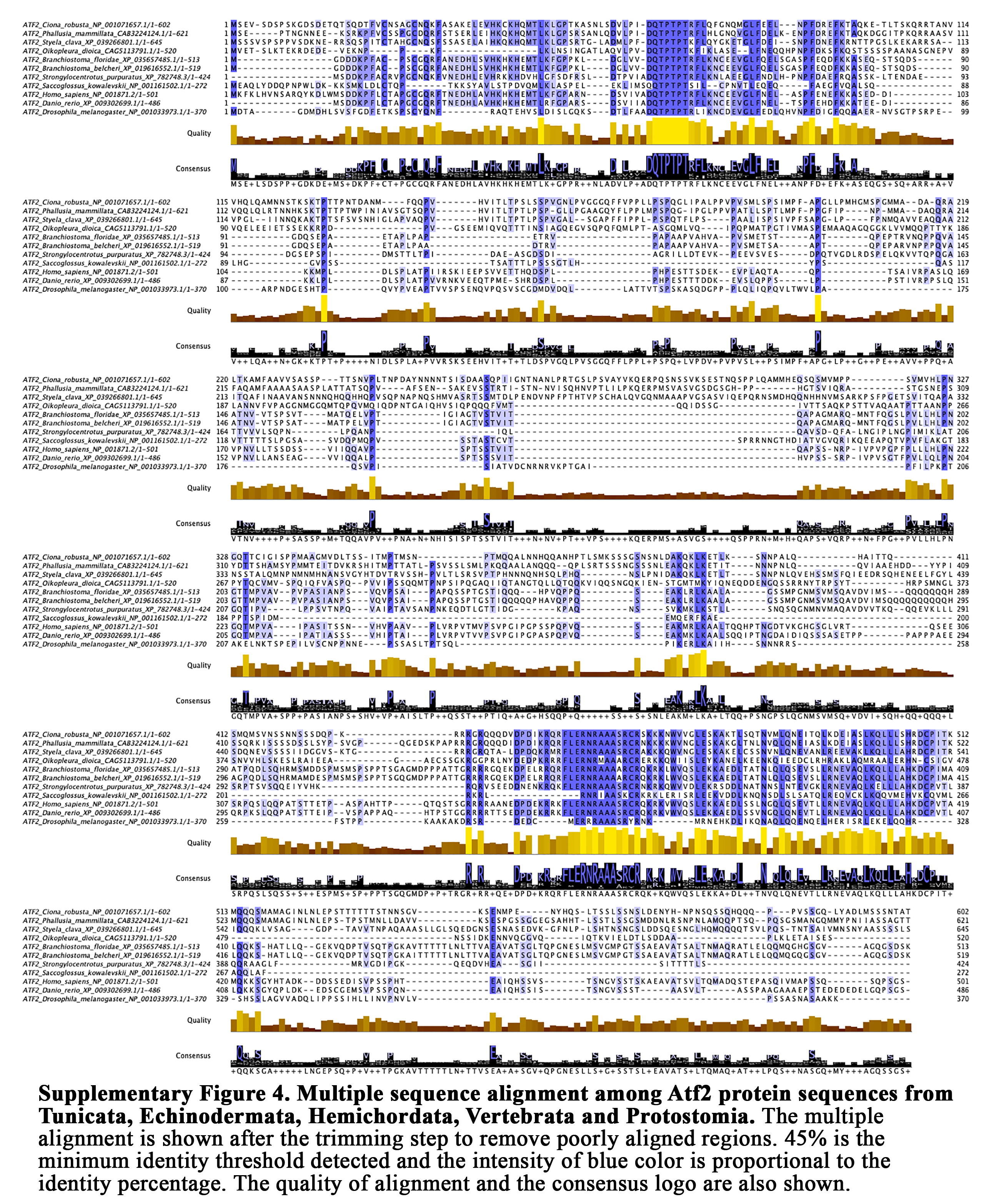

Supplement: Supplementary file 8 [file Image_4.TIF]

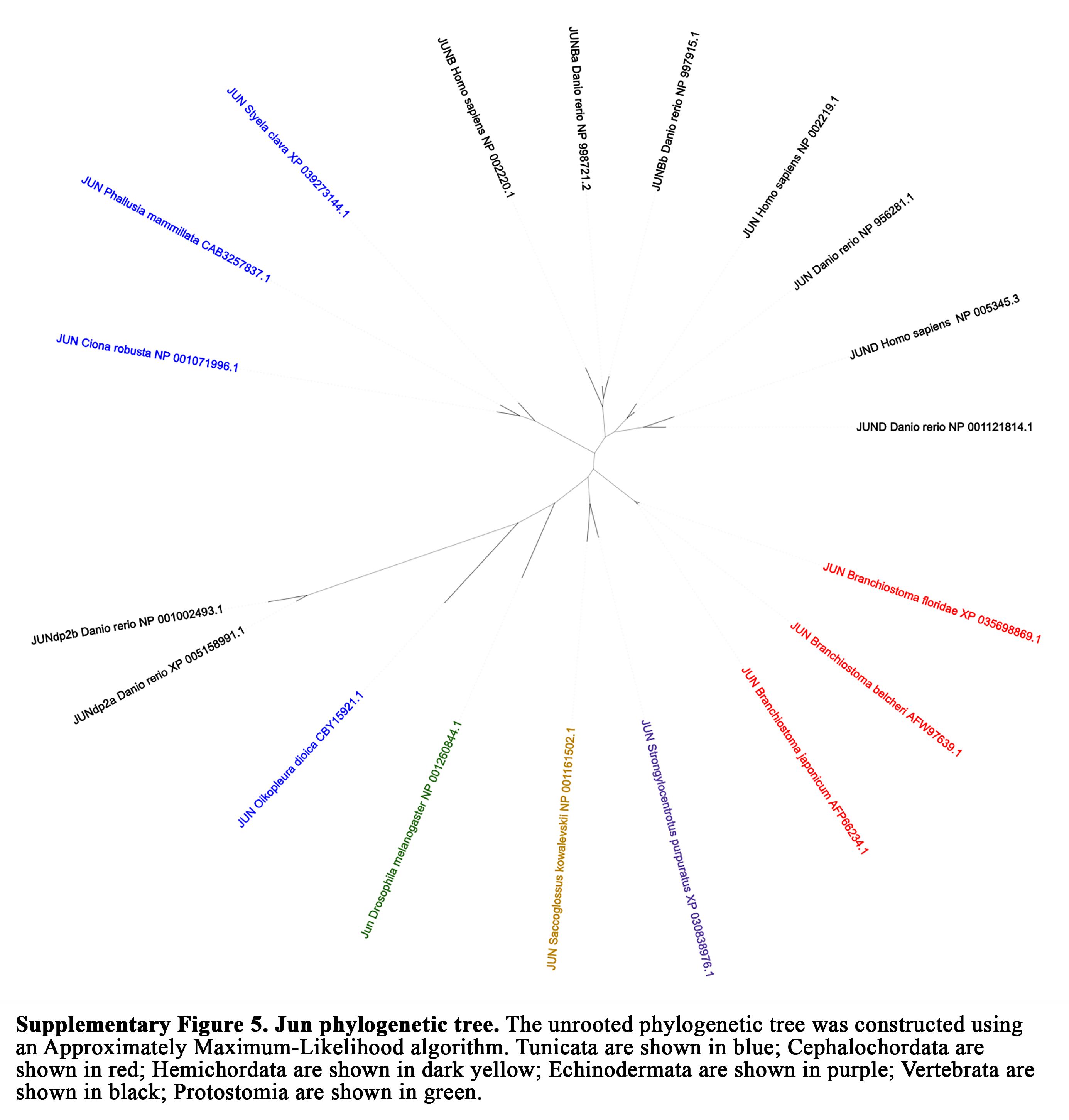

Supplement: Supplementary file 9 [file Image_5.TIF]

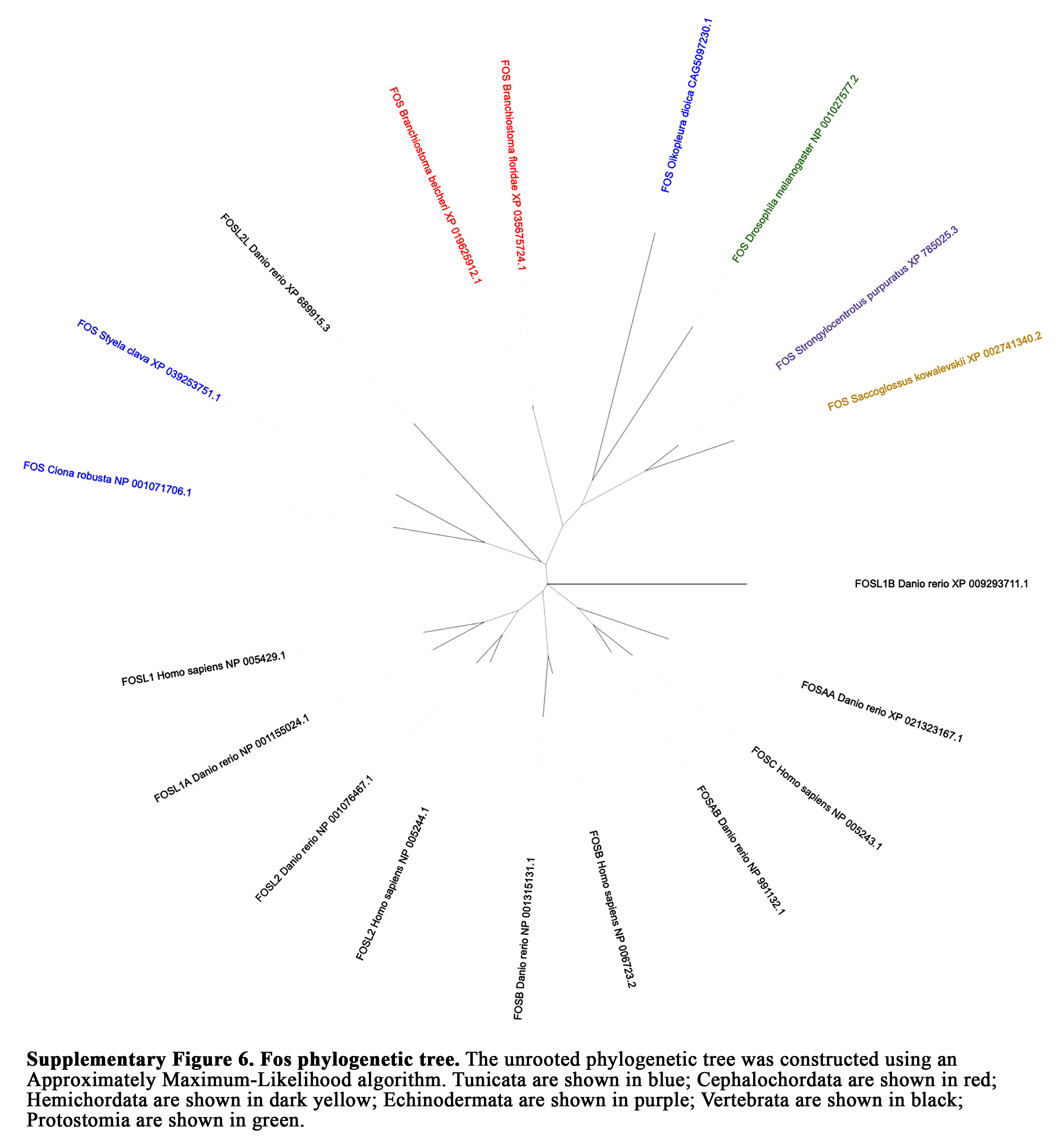

Supplement: Supplementary file 10 [file Image_6.TIF]

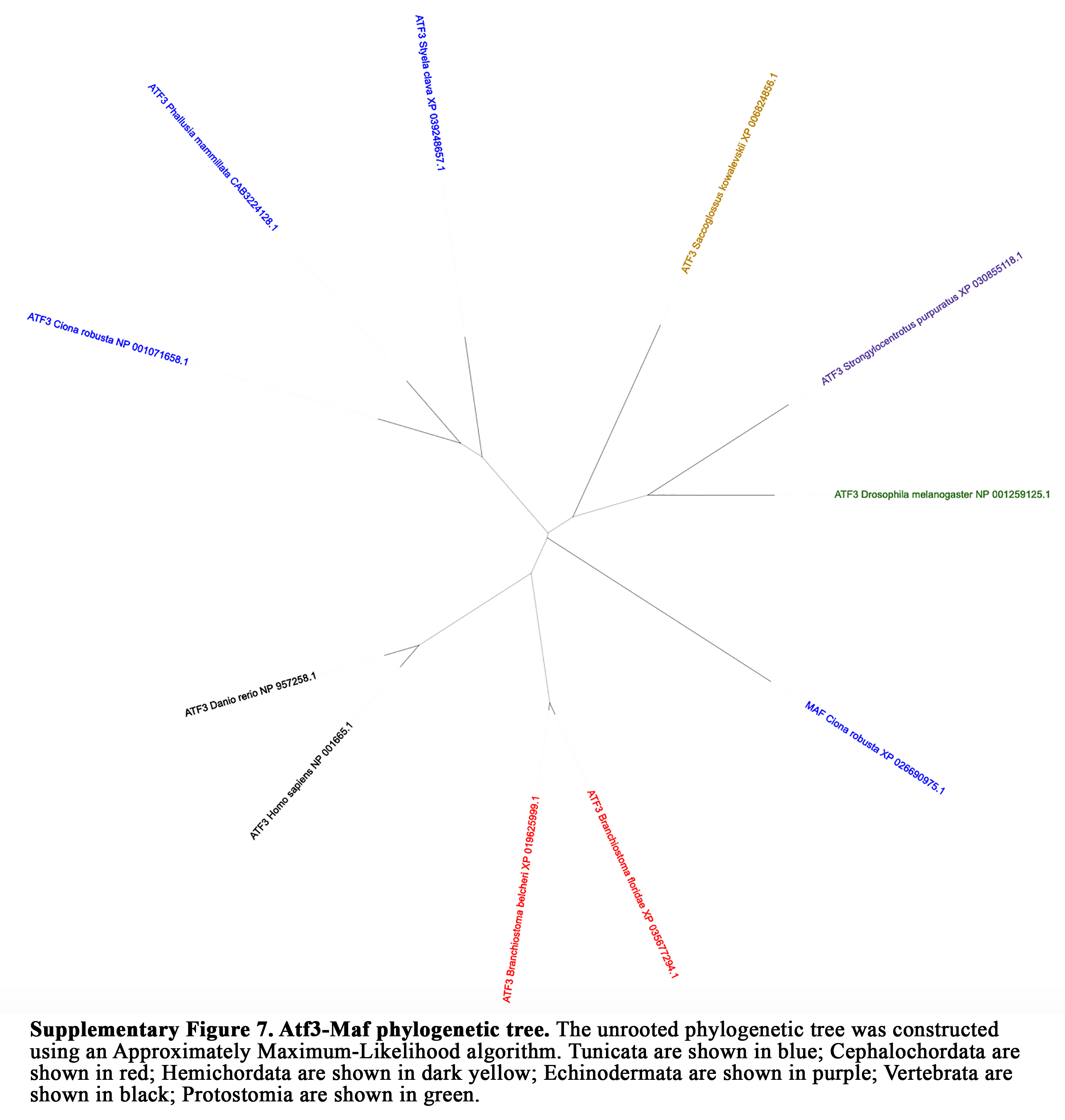

Supplement: Supplementary file 11 [file Image_7.TIF]

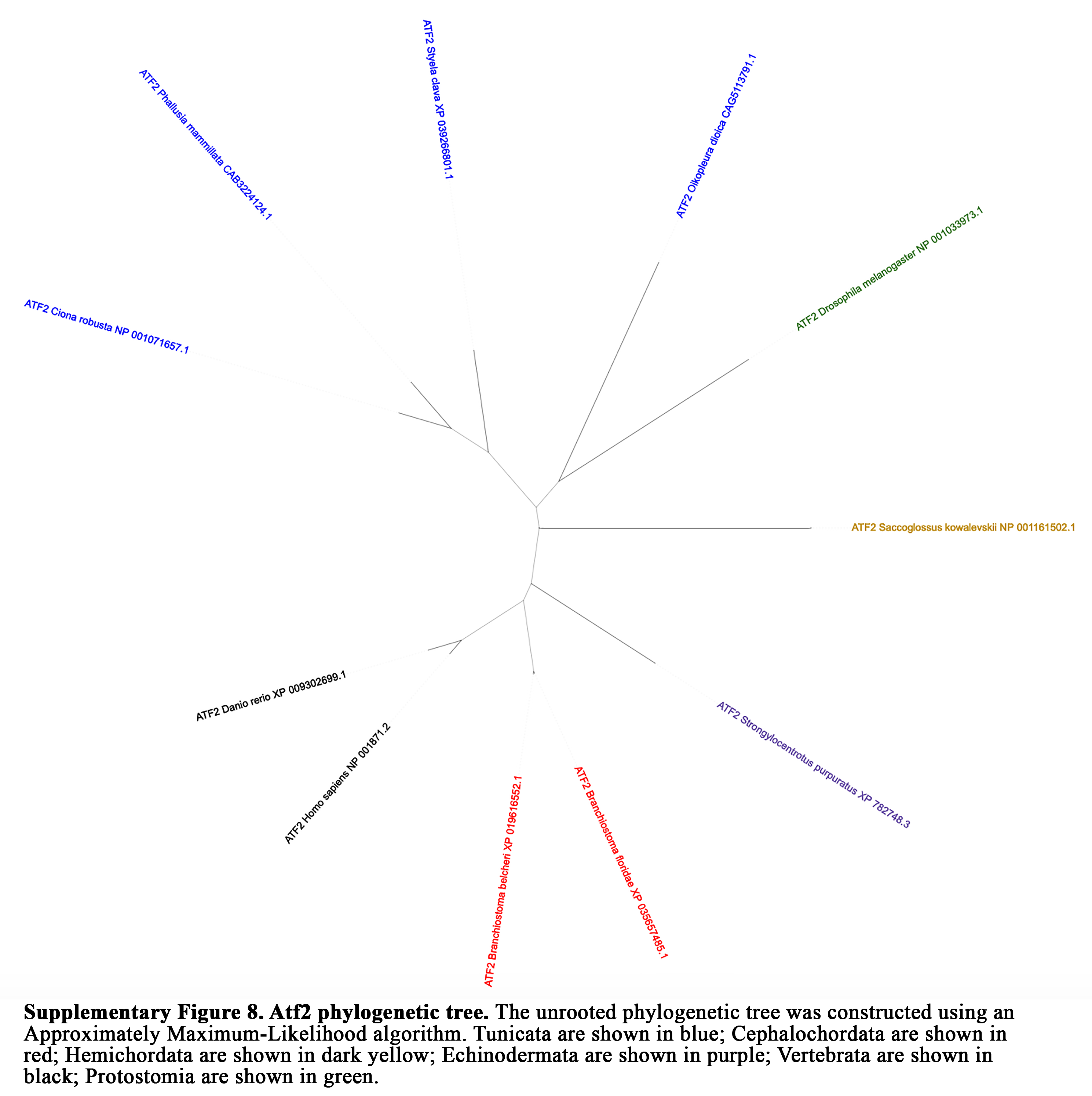

Supplement: Supplementary file 12 [file Image_8.TIF]

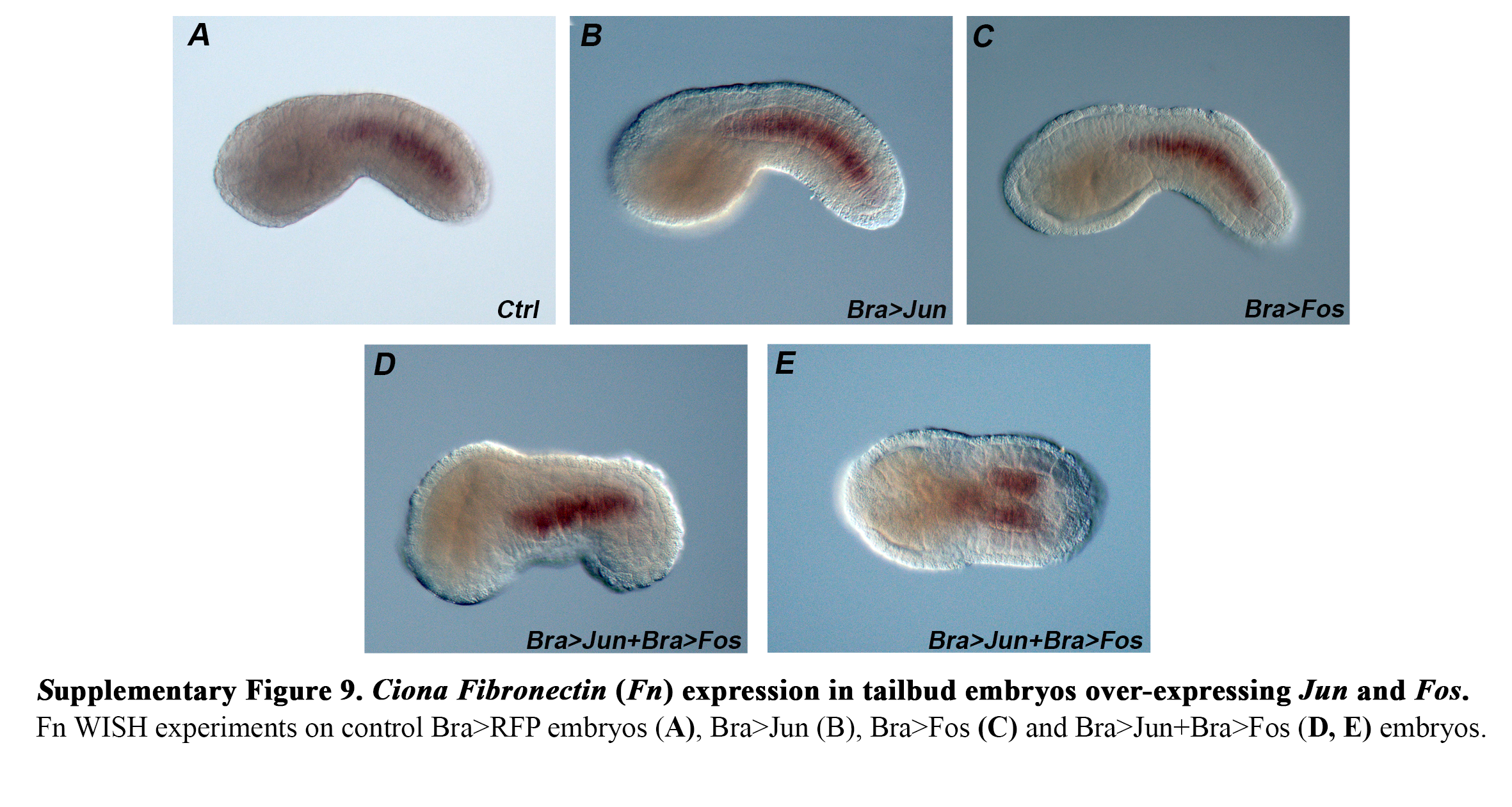

Supplement: Supplementary file 13 [file Image_9.TIF]
